# Differential diagnosis of rare adrenal cellular schwannomas: A case report

**DOI:** 10.1097/MD.0000000000037452

**Published:** 2024-03-22

**Authors:** Jiali Chen, Yan Huang, Jingjing Chen, Xianzhong Qi, Yue Ma, Miaoyan Wang, Fufeng Liu

**Affiliations:** a Department of Pathology, First People’s Hospital of Linping District, Hangzhou, China; b Department of Pathology, Taizhou People’s Hospital of Jiangsu Province, China.

**Keywords:** adrenal, adrenalectomy, cellular schwannoma, differential diagnosis, stromal tumor

## Abstract

**Background:**

Adrenal cellular schwannomas are exceptionally rare stromal tumors that are often misdiagnosed due to the lack of specific radiological, serological, or clinical features. In this report, we describe the differential diagnosis of a rare adrenal cellular schwannoma.

**Methods:**

A 69-year-old man with a history of persistent hypertension, chronic kidney disease, hypertensive heart disease, and cardiac insufficiency was hospitalized due to bilateral lower extremity edema lasting for 3 months. Plain computed tomography at that time revealed a space-occupying lesion in the right adrenal gland. As serum levels of catecholamines, cortisol, and adrenocorticotropic hormone were within normal ranges, the edema was attributed to the chronic kidney disease and cardiac insufficiency, and the patient was referred to our hospital for surgical treatment. Contrast-enhanced computed tomography revealed heterogeneous enhancement in the adrenal mass indicating pheochromocytoma. An irregularly shaped 5 cm mass with a complete capsule in the right adrenal gland was laparoscopically resected. The postoperative histopathological diagnosis was adrenal cellular schwannoma.

**Results:**

The postoperative course was unremarkable and the tumor did not recur during 5 years of follow-up.

**Conclusion:**

Adrenal cellular schwannoma is a very rare tumor that is extremely difficult to preoperatively diagnose. Histological and immunohistochemical analyses are required for differential diagnosis and confirmation. Cellular schwannomas can transform into malignant peripheral nerve sheath tumors, but not often. Consequently, regular postoperative follow-up is required for such patients, especially imaging.

## 1. Introduction

Adrenal schwannomas are benign neurogenic tumors that develop from Schwann cells in the phrenic, vagus, and sympathetic nerves.^[1]^ They are extremely rare, accounting for 0.7% of adrenal tumors and 1% to 3% of all schwannomas. Adrenal cellular schwannomas are even more uncommon.^[2]^ The lack of specific radiological, serological, and clinical features hampers preoperative diagnosis of adrenal schwannomas.^[3]^ These tumors are often preoperatively misdiagnosed as adrenocortical gland tumors or pheochromocytomas.^[4]^ Here, we describe the differential diagnosis of a rare adrenal cellular schwannoma.

## 2. Case presentation

A 69-year-old man was admitted to our hospital for surgical treatment of a space-occupying lesion in the right adrenal gland. The patient had a >10-year history of hypertension with poorly regulated blood pressure, and had been diagnosed with hypertensive heart disease, cardiac insufficiency, and chronic kidney disease within the last few years. He also had a history of diabetes for several years and had difficulty maintaining his blood sugar levels within normal limits.

**Figure 1.**
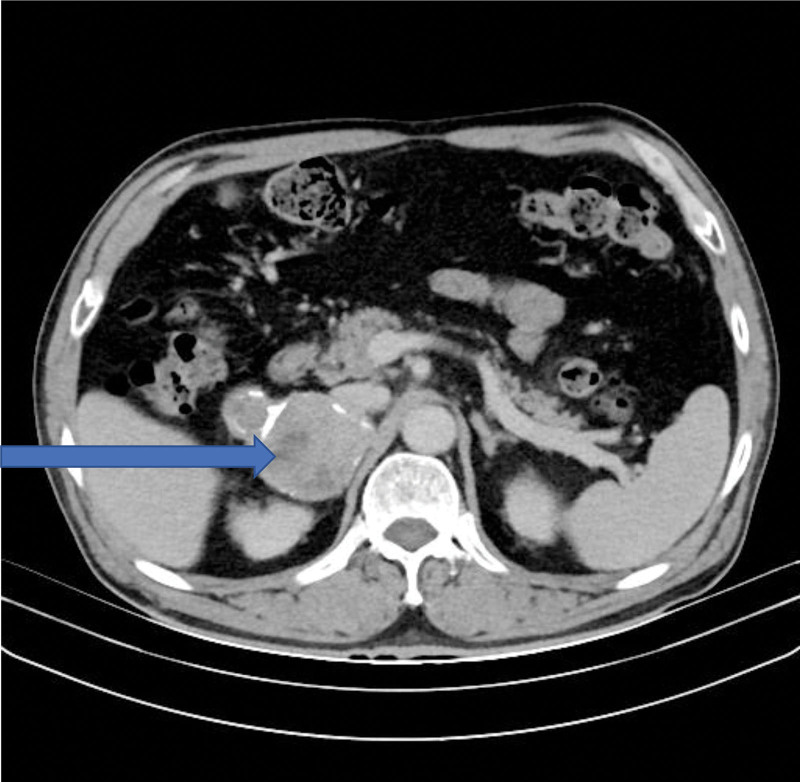
Abdominal contrast-enhanced CT image. Arrow indicates an irregular mass, measuring 5.0 × 4.8 cm, in the right adrenal gland, with progressive contrast enhancement. Nodular protrusions are evident at margin of the mass, which is surrounded by eggshell-like calcifications and bleeding is observed within it. CT = computed tomography.

He had been admitted to another hospital approximately 3 weeks before for bilateral lower extremity edema that had lasted for 3 months. Plain computed tomography (CT) revealed a space-occupying lesion with mixed density in the right adrenal gland, indicating pheochromocytoma or adrenocortical adenocarcinoma. Color duplex ultrasound of the renal arteries revealed increased resistance index in both renal arteries, with a more marked increase on the right side, which was thought to be induced by mass compression. Serum electrolyte levels, serum aldosterone-to-renin ratio, catecholamine and epinephrine levels in 24-hour urine, and a low-dose dexamethasone suppression test did not reveal any abnormalities. Hence, the lower extremity edema was attributed to the chronic kidney disease and cardiac insufficiency, and the patient was referred to our hospital for surgical treatment. Abdominal contrast-enhanced CT, performed upon admission to our hospital, revealed a 5.0 × 4.8 cm irregular mass in the right adrenal gland with increased contrast enhancement (Fig. 1). Nodules protruded at the margin of the mass, which was surrounded by eggshell-like calcifications and the inside was visibly bleeding. Pheochromocytoma was suspected, but adrenocortical adenocarcinoma could not be ruled out.

**Figure 2.**
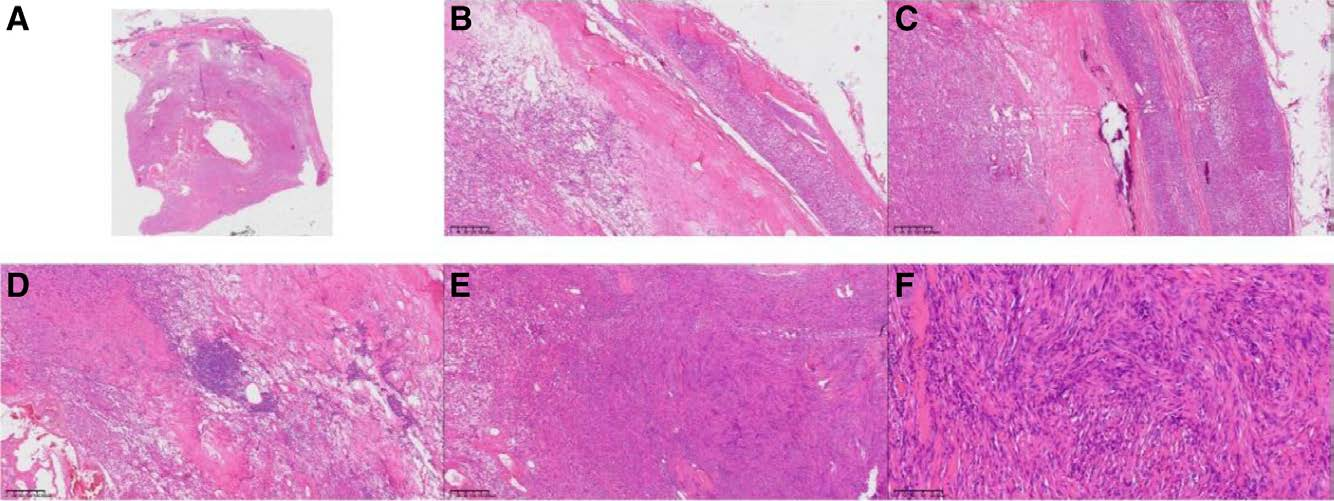
Representative histological images. (A) Panoramic view of cellular schwannoma (magnification: 10×). (B) Capsule invasion of the tumor is not evident (magnification: 40×). (C) Calcifications at the tumor margin (magnification: 40×). (D) Dilated and congested blood vessels inside the tumor, with scattered lymphoid hyperplasia (magnification: 40×). (E) Tumor mainly consists of dense spindle cells, with only Antoni A areas and no loose Antoni B areas or Verocay bodies, and no obvious necrosis (magnification: 40×). (F) The spindle cells are arranged in a spiral or striated patterns. Nuclei are spindle-shaped or oval, with a thin nuclear membrane and fine chromatin (magnification: 200×).

**Figure 3.**
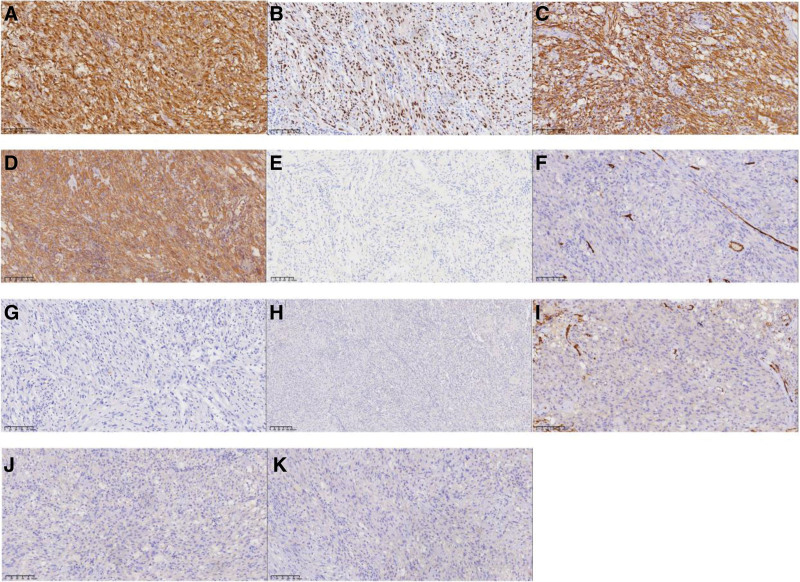
Representative images of immunohistochemical staining. Staining is positive for (A) S-100, (B) SOX10, (C) GFAP, (D) Vimentin, and (E) Ki-67 (1%), and negative for (F) CD34, (G) CD117, (H) Dog 1, (I) SMA, (J) Desmin, (K) CgA, (L) Syn, (M) HMB 45, and (N) Melan A (magnification, 200× for all). CgA = chromogranin A, GFAP = glial fibrillary acidic protein, HMB = human melanoma black, SMA = smooth muscle actin.

We laparoscopically resected an irregularly-shaped, capsulated cystic mass with a diameter of approximately 5 cm under general anesthesia. Postoperative histopathological assessment revealed well-defined boundaries and an intact capsule. Lymphoid tissue had accumulated under the capsule and within the tumor location. The tumor primarily consisted of Antoni A areas, without loose Antoni B areas and Verocay bodies, and with no signs of necrosis. The tumor comprised dense spindle cells grouped in spiral or striate patterns, with spindle-shaped or oval nuclei, a thin nuclear membrane, fine chromatin, small nucleoli, and infrequent mitotic figures. Most blood vessels in the tumor were thick-walled, with deformed lumens and intraluminal thrombi. The interstitium had scattered bleeding, calcification, and foamy histiocyte infiltration (Fig. 2). Immunohistochemical staining revealed diffuse positivity for S-100, SRY-related HMG-box 10 protein (SOX-10), glial fibrillary acidic protein, and vimentin. The Ki-67 index was approximately 1%. The tumor cells were negative for CD34, CD117, DOG-1, smooth muscle actin, desmin, chromogranin A (CgA), synaptophysin, human melanoma black-45, and Melan-A (Fig. 3). The final diagnosis was adrenal cellular schwannoma.

The patient has remained free of recurrence or malignant transformation after 5 years of follow-up.

## 3. Discussion

Schwannomas are rare spindle-cell stromal tumors that develop from Schwann cells in peripheral motor or sympathetic nerves.^[1,4]^ Although these tumors can develop at any age, the reported median age at diagnosis is 49 years, with a slight predominance of females to males of 1.2:1.^[2,5,6]^ The head and extremities are the most frequent sites of schwannomas,^[4,7]^ whereas the adrenal gland is a very uncommon location, with <100 documented cases to date.^[2,8,9]^ Adrenal schwannomas arise from Schwann cells of the phrenic, vagus, or sympathetic nerves that innervate the adrenal medulla, and account for approximately 0.7% of adrenal tumors and 1% to 3% of all schwannomas. Cellular schwannomas in the adrenal gland are even more rare with <10 cases reported to date.^[2]^

Early diagnosis is challenging, because early-stage schwannomas grow slowly and are often asymptomatic. One case series found that only 4 of 31 patients with schwannomas had clinical symptoms or elevated hormone levels.^[2]^ Another found that only 13 of 33 patients had clinical symptoms, such as abdominal or waist pain and discomfort, and only one had elevated hormone levels.^[5]^ These findings suggested that most patients with schwannomas typically do not have clinical symptoms or elevated hormone levels. Thus, adrenal schwannomas are nonsecretory tumors. Accordingly, adrenal schwannomas are very rarely considered during routine differential diagnoses. Our patient was assessed by echocardiography, adrenal B-ultrasound, and CT imaging after admission. Echocardiography revealed left ventricular diastolic dysfunction in particular. The patient’s history of persistent hypertension and a left renal cyst suggested hypertensive nephropathy. Furthermore, relevant hormone levels were within the normal range. Therefore, the bilateral lower extremity edema was attributed to chronic kidney disease and cardiac insufficiency.

Adrenocortical adenomas are mostly nonfunctioning adenomas that appear on CT as regularly shaped, space-occupying masses with uniform density in the adrenal gland area. The results of CT imaging revealed an irregularly-shaped mass with uneven density and many calcifications, which ruled out adrenocortical adenoma.^[10]^

Adrenal schwannomas with degenerative changes, such as cyst formation, calcification, and hemorrhage, appear very similar to pheochromocytomas or adrenocortical adenocarcinomas on CT images. Therefore, contrast-enhanced CT cannot distinguish among these tumors because enhancement is heterogeneous all of them.^[11,12]^ Based on the CT findings of cystic and hemorrhagic changes in the mass in our patient, we suspected pheochromocytoma, but could not rule out adrenocortical adenocarcinoma. Based on these findings, serum levels of catecholamines, adrenocorticotropic hormone, and cortisol, and the patient’s history of hypertension, our initial clinical diagnosis was nonfunctioning pheochromocytoma.

Schwannomas do not respond to chemotherapy or radiotherapy. Current guidelines recommend surgical treatment for adrenal masses >4 cm.^[13,14]^ The tumor in our patient was approximately 5 cm in diameter, which met the criteria for surgical treatment. Thus, the patient underwent laparoscopic surgery; his postoperative course was unremarkable.

In light of the challenges associated with preoperatively diagnosing adrenal schwannomas, histopathological analyses are largely the basis for a final diagnosis. The histopathological findings of the resected tumor were consistent with a cellular schwannoma, with extremely dense spindle-shaped tumor cells and no obvious nucleoli. Immunohistochemical findings indicating diffuse strong positivity for S-100, SOX10, glial fibrillary acidic protein, and vimentin further supported this diagnosis. In addition, gastrointestinal stromal tumor was excluded because the tumor cells were negative for CD117 and DOG-1. Leiomyoma was ruled out based on negativity for desmin and smooth muscle actin. The initial diagnosis of pheochromocytoma was also ruled out based on the absence of a zellballen pattern, negative CgA staining, and normal catecholamine levels. The CT appearance and histological morphology of the tumor cells did not match those of adrenocortical adenoma. Finally, infiltrative growth and prominent nucleoli typical of spindle-cell malignant melanoma were not found, and the tumor cells were negative for HMB-45 and Melan A, ruling out adrenal malignant melanoma.

Although extremely rare, transformation of schwannomas into malignant peripheral nerve sheath tumors (MPNSTs) must be considered because it is related to patient treatment and survival.^[15]^ MPNST was ruled out in our patient as we found no evidence of perineural and intraneural invasion, necrosis, mitotic figures, or heterologous components.

## 4. Conclusions

Cellular adrenal schwannomas are exceptionally rare adrenal tumors, and are therefore rarely considered in routine diagnosis and treatment. Clinicians should consider the possibility of adrenal schwannomas when CT images of patients who are clinically asymptomatic and have normal serum levels of catecholamines and other related hormones reveal an adrenal gland mass. Histopathology and immunohistochemistry play important roles in the diagnosis of adrenal schwannomas. Cellular schwannomas can transform into MPNSTs, but not often. Consequently, regular postoperative follow-up is required for such patients, especially imaging.

## Acknowledgments

We thank the radiologists, laboratory physicians, and clinicians for their assistance.

## Author contributions

**Conceptualization:** Jiali Chen, Jingjing Chen, Xianzhong Qi, Miaoyan Wang.

**Data curation:** Jiali Chen, Yan Huang, Xianzhong Qi, Yue Ma, Fufeng Liu.

**Formal analysis:** Yan Huang.

**Funding acquisition:** Fufeng Liu.

**Investigation:** Miaoyan Wang, Fufeng Liu.

**Methodology:** Yan Huang, Jingjing Chen, Xianzhong Qi, Fufeng Liu.

**Supervision:** Yue Ma.

**Writing – review & editing:** Jingjing Chen, Fufeng Liu.

**Writing – original draft:** Jiali Chen, Fufeng Liu.
